# Moderate to high levels of spin in studies comparing open and arthroscopic Latarjet procedures for anterior shoulder instability: A systematic review from the LaTour Group

**DOI:** 10.1002/ksa.70476

**Published:** 2026-06-15

**Authors:** Vasileios Sotirios Akrivos, Bertrand Claizergues, Philippe Collin, Gabriel Levy, Guillermo Vera Valencia, Floris van Rooij, Alexandre Lädermann

**Affiliations:** ^1^ Department of Orthopaedic Surgery and Musculoskeletal Trauma, Faculty of Medicine, School of Health Sciences University of Thessalia Larissa Greece; ^2^ ReSurg SA Nyon Switzerland; ^3^ Department of Orthopedic and Trauma Surgery, CHP Saint‐Grégoire Saint‐Grégoire France; ^4^ Department of Orthopedic and Trauma Surgery Clinique Victor Hugo Paris France; ^5^ Department of Orthopedic and Trauma Surgery American Hospital of Paris Neuilly‐sur‐Seine France; ^6^ Division of Orthopaedics and Trauma Surgery La Tour Hospital Meyrin Switzerland; ^7^ Department of Surgery, Division of Orthopaedics and Trauma Surgery Geneva University Hospitals Geneva Switzerland; ^8^ Faculty of Medicine University of Geneva Geneva Switzerland; ^9^ FORE (Foundation for Research and Teaching in Orthopedics, Sports Medicine, Trauma, and Imaging in the Musculoskeletal System) Meyrin Switzerland

**Keywords:** arthroscopic surgery, Latarjet procedure, reporting bias, shoulder instability, systematic review

## Abstract

**Purpose:**

The purpose of this study was to evaluate the prevalence, type and level of spin in studies comparing open and arthroscopic Latarjet procedures for anterior shoulder instability.

**Methods:**

A systematic review was conducted following Preferred Reporting Items for Systematic Reviews and Meta‐analysis (PRISMA) guidelines. PubMed was searched for comparative studies published in the last 20 years addressing open and arthroscopic Latarjet procedures. Eligible articles were screened and assessed independently by two reviewers. Spin was classified into three categories—misleading reporting, misleading interpretation and inadequate extrapolation—and graded as low, moderate or high.

**Results:**

Of the 25 included studies, 80% exhibited at least one type of spin. The most common types were selective reporting (48%), lack of discussion of limitations (20%) and claims of significant differences without statistical testing (20%). Studies with high levels of spin had a spin ratio of 1.64, while those with moderate levels had a ratio of 2.00—both higher than the 1.00 ratio observed in studies with low‐level spin. Studies with level of evidence IV showed a higher spin ratio per study. Additionally, spin was present regardless of conflict‐of‐interest declarations or funding disclosure.

**Conclusion:**

Spin is prevalent in the literature comparing open and arthroscopic Latarjet procedures, with moderate to high levels of spin more common than low. These findings highlight the importance of transparent reporting and careful interpretation of comparative surgical literature.

**Level of Evidence:**

Level IV, systematic review of Levels II–IV studies.

**Abbreviations**
ICC: intraclass correlation coefficient
JBI: Joanna Briggs Institute

## INTRODUCTION

Recurrent anterior shoulder instability with significant glenoid bone loss is a well‐recognized indication for the Latarjet procedure [2, 4, 5]. In recent years, both the open technique and the arthroscopic approach have been widely adopted, leading to ongoing discussion regarding which technique should be used [9, 16, 36].

Many studies have compared these two techniques, focusing on outcomes such as recurrence rates, healing rate and time to union, range of motion, post‐operative pain, muscle recovery, return to sport, proprioception, complication rates and graft positioning [6, 21, 24, 26, 27, 29, 32, 35, 37, 38]. However, a closer review of the literature suggests a potential issue: the presence of ‘spin’—the biased presentation or interpretation of results—which may affect how one technique is perceived as superior to the other [7, 8, 23]. Comparative studies sometimes emphasize one surgical approach over the other despite comparable reported outcomes [23]. In this context, the work by Lazarus et al. [25] provides an important framework. They developed a classification system to identify and evaluate spin in non‐randomized studies. According to their definition, spin involves ‘specific reporting strategies, either intentional or unintentional, to convince the reader that the beneficial effect of the experimental intervention in terms of efficacy and safety is greater than that shown by the results’. This methodology enables an assessment of how conclusions may misrepresent actual findings.

In addition to identifying the type of spin, this study also evaluates the level of spin, which refers to the degree of misleading interpretation in the conclusions. According to the method developed by Lazarus et al. [25], a low level of spin was defined as spin reported with uncertainty in the framing and recommendations for further trials, a moderate level as spin reported with some uncertainty in the framing or recommendations for further trials, and a high level as spin reported without any uncertainty or recommendations for further trials.

The purpose of this study was to evaluate the prevalence, type and level of spin in studies comparing open and arthroscopic Latarjet procedures for anterior shoulder instability. The primary outcome is the prevalence and type of spin. The secondary outcome is the level of spin according to established criteria. Spin was hypothesized to be present in this area of research, and that moderate to high levels of spin are more common than low‐level spin.

## METHODS

The protocol for this systematic review was submitted to PROSPERO prior to commencement (registration number CRD1028002) and conforms to the principles outlined in the handbook of the Cochrane Collaboration [14], along with the guidelines established by the Preferred Reporting Items for Systematic Reviews and Meta‐analysis (PRISMA) [31].

### Search strategy

The authors conducted a structured electronic literature search on 26 February 2025 using the PubMed database, applying the search terms (latarjet [Title] OR bone‐block [Title] OR bristow [Title]) AND (arthroscopic [Title] AND open [Title]) AND (shoulder). The search was limited to comparative articles published in the last 20 years. The start date of 2004 was selected because it approximately marks the introduction of the arthroscopic Latarjet technique into clinical practice. Since the objective of this study was to assess spin in comparative studies between the open and arthroscopic approaches, studies published before the widespread adoption of the arthroscopic technique were not relevant and therefore excluded. After removal of duplicate records, each of the two researchers screened the titles and abstracts to determine the suitability for the review using the following predefined eligibility criteria: this review included studies if they compared the outcomes between open and arthroscopic Latarjet procedures for anterior shoulder instability with no limit for sample size or prevalence. Studies were excluded if they were published in a language other than English, published before the year 2004, or were narrative, expert opinions, editorials, letters to the editor or conference proceedings. Studies reporting on animal, in vitro or in silico studies were also not considered for this review.

### Study selection

Studies that met the eligibility criteria during title and abstract screening underwent full‐text screening by two independent researchers, and any disagreement was first discussed between the researchers, and if required, a third researcher resolved any disagreement. The reference lists of studies for full‐text review were searched, and a senior expert was consulted to identify further relevant studies that may not have been captured by the database searches.

### Data extraction and quality assessment

Data extraction was performed by two researchers (V. S. A. and B. C.) independently, and their results were compared to ensure accuracy. Where there was disagreement in the documented information, the correct information was ascertained by simultaneous review of the data in question by both researchers. In the event of unresolved disagreements, a third researcher was available to make the final decision. The following data were extracted from the included studies: author(s), journal, year of publication, study design, level of evidence, conflicts of interest, funding declaration and article conclusions. If not stated within the study, the level of evidence was determined using the American Academy of Orthopaedic Surgeons recommendations [30]. Methodological quality and risk of bias of the eligible studies were assessed by two researchers according to the Joanna Briggs Institute (JBI) checklist, to appraise the reporting quality (eleven items). Where there was disagreement between the researchers, consensus was achieved by discussion and review and, if needed, the third researcher was available to make the final decision.

### Spin assessment

Spin was considered present when at least one reporting, interpretation or extrapolation strategy consistent with the classification described by Lazarus et al. [25] was identified in the study. The spin was assessed using the classification developed by Lazarus et al. [25] and was divided into three categories: (i) misleading reporting, (ii) misleading interpretation and (iii) inadequate extrapolation of the results. Each category includes specific predefined spin subcategories.

Misleading reporting was defined as incomplete or selective presentation of study results that could mislead the reader. This category included the following strategies:

(1) not reporting adverse events or lack of focus on harms,
(2) selective reporting of outcomes favouring the beneficial effect of the intervention,
(3) misleading reporting of study design,
(4) use of linguistic spin or ‘hype’,
(5) failure to consider or report study limitations and
(6) selective citation of other studies.

Misleading interpretation was defined as interpretation of results in a way that overestimates the beneficial effect of the intervention. This category included:

(1) claiming a beneficial effect despite statistically non‐significant results,
(2) claiming equivalence between interventions despite non‐significant results with wide confidence intervals,
(3) claiming safety despite non‐significant safety outcomes and insufficient statistical power,
(4) claiming a difference between groups despite absence of appropriate statistical testing,
(5) focusing on statistical significance without consideration of clinical relevance and
(6) use of causal language or causal claims in non‐randomized studies.

Inadequate extrapolation was defined as inappropriate generalization of study findings beyond the study context. This category included:

(1) extrapolation of results to a broader population, intervention or outcome than those studied and
(2) inappropriate or unsupported implications for clinical practice.

Each included study was evaluated for the presence of one or more of these predefined spin strategies. Multiple spin strategies could be identified within a single study. The two researchers independently identified and analysed the presence of spin in the selected articles, and the third researcher was consulted in case of disagreement.

In addition to the type of spin, the two researchers evaluated the level of spin from the conclusions of the studies, according to the method developed by Lazarus et al. [25]. A low level of spin was defined as spin reported with uncertainty in the framing and recommendations for further trials, a moderate level as spin reported with some uncertainty in the framing or recommendations for further trials, and a high level as spin reported without any uncertainty or recommendations for further trials. Any discrepancies were solved by consensus and, if needed, by consultation with the third researcher.

The level of spin reflects the framing of study conclusions, whereas the spin ratio quantifies the number of distinct spin strategies identified per study; both measures were evaluated to capture complementary aspects of reporting bias. To minimize subjectivity, all spin assessments were independently performed by orthopaedic surgeons with subspecialty training in shoulder surgery.

### Statistical analysis

The data were reported as frequencies and proportions. No statistical analysis was performed. In addition to overall prevalence, spin was descriptively analysed according to study characteristics, including level of evidence, conflict of interest disclosure, funding status and year of publication. The spin ratio was defined as the total number of identified spin instances divided by the number of studies within a given subgroup (e.g., studies with declared funding or specific conflict‐of‐interest status) and was used to describe the frequency of spin across study characteristics. The spin ratio represents the average number of spin instances per study within a given subgroup and was calculated by dividing the number of identified spin instances by the number of studies in that subgroup. This metric was used to describe the frequency of spin across different study characteristics.

## RESULTS

The systematic search returned 79 records, and 48 studies were excluded by examining their titles and/or abstracts, and a further six studies were excluded after full‐text review, which left 25 eligible studies (Table 1, Figure 1) [1, 6, 10, 11, 13, 15, 16, 17, 18, 20, 21, 22, 24, 26, 27, 28, 29, 32, 33, 34, 35, 36, 37, 38, 39]. Inter‐observer agreement for the assessment of spin according to Lazarus et al. was excellent (intraclass correlation coefficient [ICC], 1.00).

**Table 1:** Summary of included studies comparing open versus arthroscopic Latarjet procedures. Information includes author, year, study design, level of evidence, conflict of interest declaration and funding status.

Author	Year	Journal	Study design		Level of evidence	Conflict of interest reported?	Conflict of interest	Funding
Nascimento	2024	*JSES*	Retrospective	Comparative	III	No	The authors, their immediate families, and any research foundations with which they are affiliated have not received any financial payments or other benefits from any commercial entity related to the subject of this article.	No funding was disclosed by the authors.
Lee	2024	*Arthroscopy*	Systematic review		IV	Yes	A. E. J. has received funding grants from Arthrex. All other authors (M. S. L., S. M. P., T. K., J. M., N. P., R. J. M. S. F., S. M. G., A. D., S. S., A. G.) declare that they have no known competing financial interests or personal relationships that could have appeared to influence the work reported in this paper.	
Lallemand	2024	*JSES*	Retrospective	Comparative	III	Yes	Laurent Lafosse has received consultancy fees from Johnson & Johnson DePuy Synthes, Stryker, and Smith & Nephew and royalties from Johnson & Johnson DePuy Synthes. Thibault Lafosse has received consultancy fees from Johnson & Johnson DePuy Synthes, Stryker and Smith & Nephew. Geert A. Buijze receives consultancy fees from Stryker. The other authors, their immediate families and any research foundations with which they are affiliated have not received any financial payments or other benefits from any commercial entity related to the subject of this article.	No funding was disclosed by the authors.
Tanaka	2024	*JSES*	Retrospective	Comparative	III	No	The authors, their immediate families and any research foundations with which they are affiliated have not received any financial payments or other benefits from any commercial entity related to the subject of this article.	No funding was disclosed by the authors.
Vuletić	2024	*Curr Rev Musculoskelet Med*	Review		IV	Yes	The authors declare no competing interests. F. V. declares he has no competing interests. B. B. has received payment for lectures/faculty involvement from S + N and Ortomedic and is on the Board/Editorial Board of *ESSKA*, *JISAKOS*, *KSSTA*, *AJSM* and *VJSM*	
Orgiu	2024	*Orthop Traumatol Surg Res*	Prospective	Comparative	III	Yes	A. O. received compensation from the company Smith & Nephew; BB received compensation from the companies Corin, Sanofi and Coloplast; H. B. received compensation from the companies LEO Pharma, Sanofi, Smith & Nephew, Orthofix, Exafield, SI‐Bone, B3TSI, Colopast, Bva, Arthrex; M. A. R. received compensation from the companies, Corin, FH‐Orthopedics LEO Pharma, Sanofi, Digital Medical Hub, Johnson & Johnson Medical, Spine Innovations, Pfizer; P. B. received compensation from the companies, Arthrex, Depuy, FX Solutions LEO Pharma, Sanofi, Johnson & Johnson Medical, Smith & Nephew; and P. R. did not receive compensation from any company.	This study did not benefit from any funding source.
Lacouture‐Suarez	2023	*Arthrosc Sports Med Rehabil*	Systematic review		IV	Yes	P. B. reports personal fees and other from Smith & Nephew, outside the submitted work. Full ICMJE author disclosure forms are available for this article online, as supplementary material.	
Deng	2023	*Orthop J Sports Med*	Systematic review Meta‐analysis	Comparative	III	No	This study was supported by the Guangdong Basic and Applied Basic Research Foundation (2021A1515220030), the Shenzhen Science and Technology Program (RCBS20200714114856299, RCYX20210609103902019) and the Clinical Research Project of Shenzhen Second People's Hospital (20203357038). AOSSM checks author disclosures against the Open Payments Database (OPD). AOSSM has not conducted an independent investigation on the OPD and disclaims any liability or responsibility relating thereto.	
Maiotti	2023	*JSES*	Retrospective	Comparative	III	No	The authors, their immediate families and any research foundation with which they are affiliated have not received any financial payments or other benefits from any commercial entity related to the subject of this article	No funding was disclosed by the authors.
Girard	2022	*Am J Sports Med*	Retrospective	Comparative	III	Yes	V. M. has received consulting fees from SBM (Science & BioMaterials). N. B. has received consulting fees from SBM & Move up. AOSSM checks author disclosures against the Open Payments Database (OPD). AOSSM has not conducted an independent investigation on the OPD and disclaims any liability or responsibility relating thereto.	
Hurley	2021a	*Arthrosc Sports Med Rehabil*	Retrospective	Comparative	III	Yes	L. M. J. has received grants from Arthrex, Mitek and Smith & Nephew, outside the submitted work. Full ICMJE author disclosure forms are available for this article online, as supplementary material.	
Bonnevialle	2021	*Am J Sports Med*	Retrospective	Comparative	III	Yes	N. B. has received fees for participating in an education program with Smith & Nephew. V. M. has received royalties from SBM. AOSSM checks author disclosures against the Open Payments Database (OPD). AOSSM has not conducted an independent investigation on the OPD and disclaims any liability or responsibility relating thereto.	
Hurley	2021b	*Knee Surg Sports Traumatol Arthrosc*	Retrospective	Comparative	III	Yes	Laith Jazrawi reports research support from Arthrex, Mitek and Smith & Nephew, unrelated to this study. Mandeep Virk reports personal fees from Acumed and Exactech, unrelated to this study. Robert Meislin reports personal fees from Arthrex, CONMED, Mitek and Stryker, unrelated to this study. None of the other authors report any conlict of interest.	No funding was received for this study.
Ali	2020	*Arthroscopy*	Retrospective	Comparative	III	No	The authors report no conflicts of interest in the authorship and publication of this article. Full ICMJE author disclosure forms are available for this article online, as supplementary material.	
Malahias	2019	*Eur J Orthop Surg Traumatol*	Systematic review		III	Not reported		
Hurley	2019	*Am J Sports Med*	Systematic review Meta‐analysis		III	No	The authors declared that they have no conflicts of interest in the authorship and publication of this contribution.	
Neyton	2018	*Arthroscopy*	Retrospective	Comparative	III	Yes	L. N. receives support from Tornier Wright, Arthrex. P. B. receives support from Smith & Nephew, Wright. Imascap. Equity. G. W. receives support from Tornier Wright, Imascap. L. L. receives support from DePuy, Stryker, DePuy Mitek.	
Horner	2018	*BMC Musculoskelet Disord*	Systematic review		IV	Yes	O. A. is an editorial board member of the journal of *BMC Musculoskeletal Disorders.*	No funding was received for the completion of this project.
Zhu	2017	*Am J Sports Med*	Prospective	Comparative	III	No	Y. Z. was sponsored by the training program for high‐level technicians in the health care system of Beijing (code: 2014‐3‐028) for this study. C. J. was sponsored by Beijing Municipal Administration of Hospitals Clinical Medicine Development of Special Funding (code: XMLX201511) for this study.	
Kordasiewicz	2017	*Int Orthop*	Retrospective	Comparative	III	Not reported		
Marion	2017	*Arthroscopy*	Prospective	Comparative	II	Yes	J. D. receives support from Mitek. T. B. receives support from Arthrex France. P. H. receives support from Arthrex France, Zimmer and Medacta.	
Russo	2017	*Joints*	Prospective	Comparative	II	Not reported		
Nourissat	2016	*Orthop Traumatol Surg Res*	Prospective	Comparative	II	No	The authors declare that they have no competing interest.	
Cunningham	2016	*Knee Surg Sports Traumatol Arthrosc*	Retrospective	Comparative	III	No	The authors present no conflicts of interest.	
Randelli	2016	*Knee Surg Sports Traumatol Arthrosc*	Systematic review		III	Not reported		

**Figure 1:**
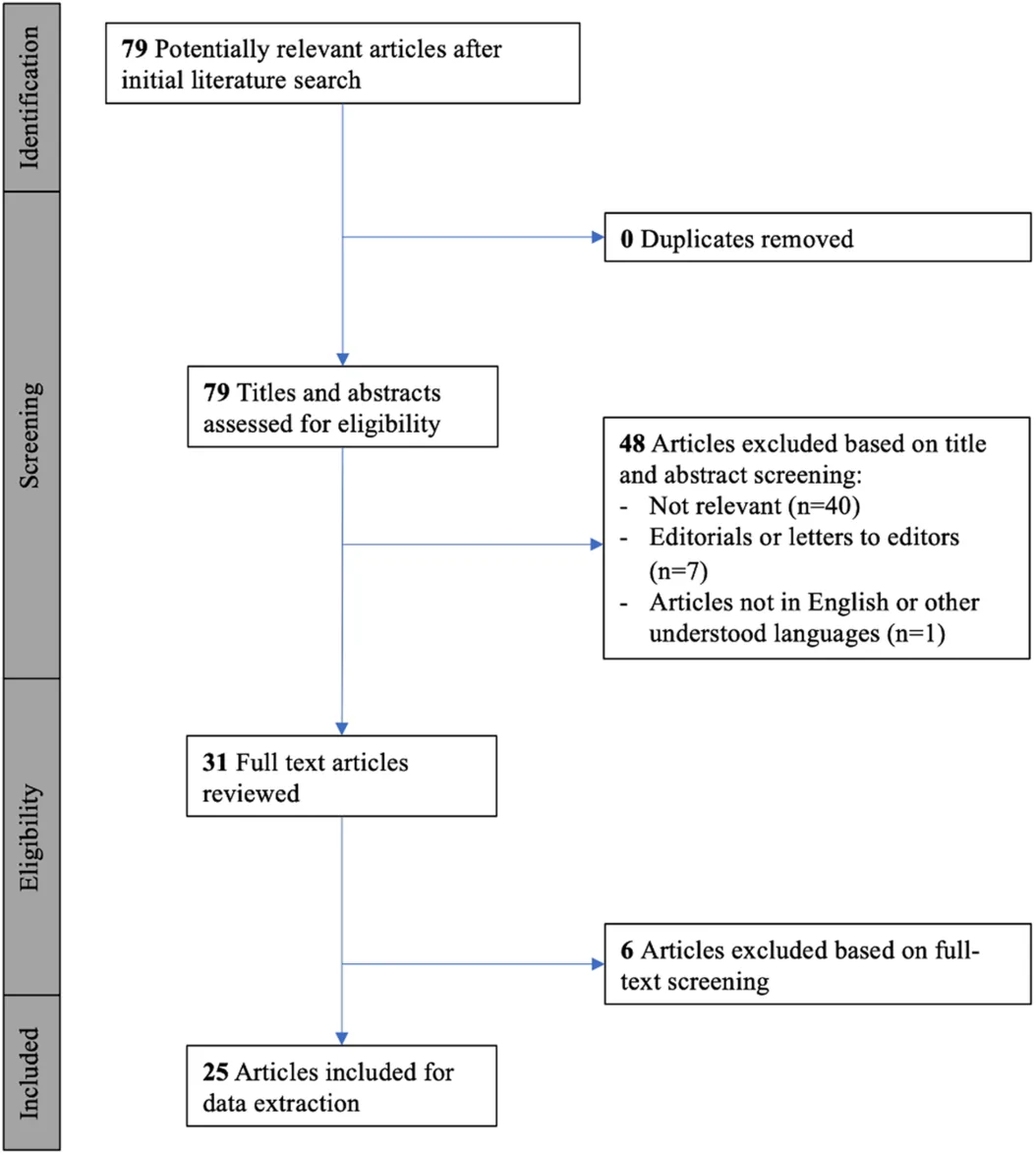
PRISMA flow diagram showing the study selection process. Outlines the number of records identified, screened, excluded and included in the final systematic review (*N* = 25). PRISMA, Preferred Reporting Items for Systematic Reviews and Meta‐Analyses.

### Risk of bias

Of the 25 studies, five were prospective comparative studies, 12 were retrospective comparative studies, five were systematic reviews, two were systematic reviews with meta‐analysis, and one was a review. For the eight reviews, publication bias was not assessed in 88% of studies, and recommendations for policy and/or clinical practice were not supported by the reported data in 75% of studies (Table 2). For the 17 clinical studies, strategies to deal with confounding factors were not stated in 82% of studies, and there were no strategies to address incomplete follow‐up utilized in 88% of studies (Table 3).

**Table 2:** Risk of bias assessment for review articles included in the analysis (*N* = 8).

Systematic reviews		Lee, 2024	Vuletić, 2024	Lacouture‐Suarez, 2023	Deng, 2023	Malahias, 2019	Hurley, 2019	Horner, 2018	Randelli, 2016
1	Is the review question clearly and explicitly stated?	Y	N	Y	Y	Y	Y	Y	Y
2	Were the inclusion criteria appropriate for the review question?	Y	NA	Y	Y	Y	Y	Y	Y
3	Was the search strategy appropriate?	Y	NA	Y	Y	Y	Y	Y	Y
4	Were the sources and resources used to search for studies adequate?	Y	NA	Y	Y	Y	Y	Y	Y
5	Were the criteria for appraising studies appropriate?	Y	NA	Y	Y	Y	Y	Y	Y
6	Was critical appraisal conducted by two or more reviewers independently?	Y	NA	Y	Y	Y	Y	Y	N
7	Were there methods to minimize errors in data extraction?	Y	NA	Y	Y	Y	Y	Y	N
8	Were the methods used to combine studies appropriate?	Y	Y	Y	Y	Y	Y	Y	Y
9	Was the likelihood of publication bias assessed?	N	N	N	Y	N	N	N	N
10	Were recommendations for policy and/or practice supported by the reported data?	Y	N	N	Y	N	N	N	N
11	Were the specific directives for new research appropriate?	Y	Y	Y	Y	Y	Y	Y	Y

*Note*: Evaluated using the Joanna Briggs Institute (JBI) checklist.

Abbreviations: N, no; NA, not applicable; Y, yes.

**Table 3:** Risk of bias assessment for cohort studies (*N* = 17).

Cohort studies		Nascimento, 2024	Lallemand, 2024	Tanaka, 2024	Orgiu, 2024	Maiotti, 2023	Girard, 2022	Hurley, 2021	Bonnevialle, 2021	Hurley, 2021	Ali, 2020	Neyton, 2018	Zhu, 2017	Kordasiewicz, 2017	Marion, 2017	Russo, 2017	Nourissat, 2016	Cunningham, 2016
1	Were the two groups similar and recruited from the same population?	Y	N	Y	U	Y	Y	Y	Y	Y	N	Y	Y	N	Y	Y	Y	Y
2	Were the exposures measured similarly to assign people to both exposed and unexposed groups?	Y	N	Y	Y	Y	Y	Y	Y	Y	Y	Y	Y	Y	Y	Y	Y	Y
3	Was the exposure measured in a valid and reliable way?	Y	Y	Y	Y	Y	Y	Y	Y	Y	Y	Y	Y	Y	Y	Y	Y	Y
4	Were confounding factors identified?	Y	Y	U	Y	Y	Y	Y	Y	Y	Y	Y	Y	Y	Y	Y	Y	Y
5	Were strategies to deal with confounding factors stated?	Y	N	N	Y	Y	N	N	N	N	U	N	U	N	N	N	N	N
6	Were the groups/participants free of the outcome at the start of the study (or at the moment of exposure)?	Y	U	Y	Y	Y	Y	Y	Y	Y	Y	Y	Y	Y	Y	Y	Y	Y
7	Were the outcomes measured in a valid and reliable way?	Y	Y	Y	Y	Y	Y	Y	N	Y	Y	Y	Y	Y	Y	Y	Y	Y
8	Was the follow‐up time reported and sufficient to be long enough for outcomes to occur?	U	U	Y	U	U	U	U	U	U	U	Y	U	U	U	Y	U	U
9	Was follow‐up complete, and if not, were the reasons to loss to follow‐up described and explored?	Y	N	Y	U	U	U	U	Y	Y	Y	N	N	Y	Y	Y	N	Y
10	Were strategies to address incomplete follow‐up utilized?	NA	N	Y	Y	U	N	N	NA	NA	N	N	N	N	N	NA	N	NA
11	Was appropriate statistical analysis used?	Y	Y	U	U	U	Y	Y	Y	Y	Y	Y	Y	N	Y	Y	U	N

*Note*: Evaluated using the JBI checklist.

Abbreviations: JBI, Joanna Briggs Institute; N, no; NA, not applicable; U, unknown; Y, yes.

### Prevalence of spin in included studies

Of the 25 studies, 20 had at least one type of spin (80%). Of the misleading reporting, the most common type was ‘selective reporting’ (48%), followed by ‘no consideration of the limitations’ (20%) and ‘not reporting adverse events or lack of focus on harm’ (8%) (Table S1). Of the inadequate interpretation, the most common type was ‘claim of any significant difference despite lack of statistical test’ (20%), followed by ‘claim an equivalence for non‐statistically significant results despite a wide confidence interval’ (8%), and ‘causal language or causal claim’ (8%). Of the inadequate extrapolation, the most common type was ‘inadequate extrapolation to larger population, intervention or outcome’ (8%), followed by ‘inadequate implication for clinical practice’ (4%).

### Prevalence of spin per level of evidence

The three studies of Level II had a ratio of spin per study of 1.00 (Table S2), the 18 studies of Level III had 1.17, while the four studies of Level IV had 2.00.

### Prevalence of spin per conflict of interest

Of the 25 studies, twelve declared their conflicts of interest, nine declared that they had no conflict of interest, and four did not report it (Table S3). The twelve studies that reported their conflicts of interest had a ratio of spin per study of 1.25, the nine studies that declared they had no conflict of interest had a ratio of 1.22, and the four that did not report it had a ratio of 1.50.

### Prevalence of spin per funding

Of the 25 studies, none of them reported any funding, seven declared that they received no funding, and 18 did not report it (Table S4). The seven studies that declared they received no funding had a ratio of spin per study of 1.29, and the 18 that did not report it had a ratio of 1.28.

### Level of spin

Of the 25 studies, eleven had a high level of spin, five had a moderate level, and four had a low level of spin (Table S5). The 11 studies with a high level of spin had a ratio of spin per study of 1.64, the 5 studies with a moderate level of spin had a ratio of 2.00, and the 4 studies with a low level had a ratio of 1.00.

### Prevalence of spin by risk of bias and publication date

Descriptive comparison of spin frequency across study characteristics is presented according to assessed risk of bias and publication year. Studies with a higher assessed risk of bias (60%–79%) exhibited a greater frequency of spin, with 15 instances identified across 13 studies (spin ratio per study = 1.15), compared to 9 instances across 8 studies (spin ratio = 1.13) among those with a lower risk of bias (80%–100%) (Table S6). When stratified by publication date, spin appeared in recent publications (2023–2025), with 11 instances across 9 studies (spin ratio = 1.22), while earlier studies (2020–2022) had only 4 instances across 5 studies (spin ratio = 0.80) (Table S7).

## DISCUSSION

The main finding of this study is that spin was present in 80% (20 out of 25) of the included studies, confirming the hypothesis that spin is found in this area of research. Moreover, most of these studies demonstrated moderate to high levels of spin, suggesting a tendency for authors to present findings in a way that aligns with their interpretation or perspective, which may reflect inherent bias rather than intentional.

The most frequent forms of spin included selective reporting (48%), ignoring study limitations (20%) and claiming significant differences without proper statistical testing (20%). These findings may reflect challenges in reporting and interpreting comparative surgical outcomes, particularly in studies with methodological limitations. Differences between reported conclusions and underlying data may arise from variability in study design, statistical power and interpretation of results. A lack of statistical knowledge, the desire to support a preferred technique, or even the expectation that stronger conclusions will make a study more likely to be published. These interpretations are speculative and cannot be directly inferred from the data. As a result, spin can appear in both the presentation and interpretation of results, either by overstating differences or by suggesting equivalence or no difference when the available evidence may not be sufficient to support such conclusions.

The presence of spin has meaningful implications for clinical decision‐making and scientific integrity. Both the open and arthroscopic techniques are widely accepted, but when study results are presented in a misleading way, it can give a false impression of which method is better. This may lead surgeons to choose a technique based on the author's conclusions rather than on solid evidence. This underscores the importance for both clinicians and researchers to critically evaluate the evidence and not solely rely on the abstract or conclusion when interpreting findings. An important consideration raised by this study is the potential direction of spin, specifically whether conclusions preferentially favour open or arthroscopic techniques and whether such trends evolve over time. Although evaluating directional spin could provide insight into whether reporting strategies align with surgeon preferences or prevailing practice trends, this was not assessed in the present study. The Lazarus framework focuses on the presence and framing of spin rather than its direction, and determining directional bias would require additional subjective interpretation beyond the scope of the predefined methodology.

When spin was classified according to the Lazarus level classification, studies with a high level of spin had a spin ratio of 1.64, those with a moderate level had a spin ratio of 2.00, while studies with a low level of spin had a ratio of 1.00. In this descriptive analysis, studies classified as having moderate or high levels of spin demonstrated a higher number of spin instances per study compared with those classified as having low‐level spin. Given that most included studies were Level III or IV evidence, the findings should be interpreted in the context of known methodological limitations inherent to these study designs. These studies may rely more on spin to offset methodological limitations. As such, their conclusions require careful interpretation, and stronger reporting standards are needed to limit bias. Reviews were included because they synthesize and interpret primary data, are frequently cited and strongly influence clinical understanding; as such, they may also propagate or amplify reporting and interpretive spin.

In this descriptive analysis, Level IV studies demonstrated the highest spin ratio per study. This is likely due to the inherent weaknesses in study design, which can make it easier for authors to present results more positively than the data justify. When spin is added onto these studies, it can significantly alter the results and potentially mislead clinicians and readers. Similar spin ratios were descriptively observed regardless of conflict‐of‐interest or funding disclosure; however, no statistical testing was performed, and no conclusions regarding the effect of disclosure on spin can be drawn.

Several studies have evaluated the presence of spin in systematic reviews related to treatments for massive irreparable rotator cuff tears. Fathi et al. [12] found spin in 27 of 29 studies (93.1%) reviewing biodegradable balloon spacers, a higher rate than in our study (80%), with a tendency for spin in abstracts to favour the balloon spacer intervention. In another study by Bashrum et al. [3], 53.8% (7/13) of systematic reviews on tendon transfers for irreparable rotator cuff tears contained at least one form of spin in the abstract. The most common type was Type 5 spin—where conclusions claimed a beneficial effect of the treatment despite a high risk of bias in primary studies—found in 23.1% (3/13) of abstracts. This differs from our study, where selective reporting was the most common form of spin (48%). Similarly, Kim et al. [19] reported that all 17 included studies (100%) on superior capsular reconstruction exhibited at least one form of spin, again exceeding the 80% rate found in our analysis. Their review identified an association between the presence of spin and lower levels of evidence. Our findings align with this trend, reinforcing the concern that spin tends to occur more frequently in studies with lower levels of evidence across various interventions in the shoulder surgery field. Kim et al. [19] also observed that spin was more common in more recent publications, particularly within the category of misleading interpretation. All analyses were descriptive, and no statistical comparisons were performed; therefore, observed differences should not be interpreted as causal associations.

An important consideration is whether any level of spin can be considered acceptable in scientific reporting. Currently, there are no established thresholds defining acceptable levels of spin. Even low levels of spin may influence reader interpretation. Therefore, spin should not be considered acceptable but rather minimized through transparent and accurate reporting. The classification of spin into low, moderate and high levels in this study is intended to describe the degree of distortion in interpretation rather than to imply that certain levels are permissible. Readers should interpret study findings cautiously, particularly when moderate or high levels of spin are present.

### Limitations

This study has several limitations. First, assessment of spin is inherently interpretive and, despite use of a validated framework and excellent interobserver agreement, may remain subject to some degree of subjectivity. Second, all analyses were descriptive, and no inferential statistical testing was performed; therefore, observed differences in spin prevalence or spin ratios across study characteristics should not be interpreted as statistically significant associations. Third, the included studies largely lacked high‐level evidence, with most classified as Level III or IV, which are more prone to methodological limitations. Fourth, the study did not evaluate the direction of spin or whether conclusions favoured one surgical technique over the other, as this was beyond the scope of the predefined classification framework. Finally, inclusion of both primary studies and review articles may have introduced heterogeneity, and although the Lazarus classification provides a structured approach, it may not capture all possible forms of spin.

## CONCLUSION

Spin is highly prevalent in the literature comparing open and arthroscopic Latarjet procedures, with moderate to high levels of spin more common than low. These findings highlight the importance of transparent reporting and careful interpretation of comparative surgical literature.

## AUTHOR CONTRIBUTIONS

Vasileios S. Akrivos and Bertrand Claizergues contributed to literature search, data extraction, spin assessment and drafting of the manuscript. Philippe Collin, Floris van Rooij and Alexandre Lädermann contributed to study design, methodological supervision and critical revision of the manuscript. Gabriel Levy and Guillermo Vera Valencia contributed to data interpretation and manuscript revision. Alexandre Lädermann provided senior supervision. All authors reviewed and approved the final manuscript.

## CONFLICT OF INTEREST STATEMENT

Alexandre Lädermann is a paid consultant for Arthrex, Stryker and Medacta. He received royalties from Stryker and Medacta. He is the (co‐)founder of FORE, Med4Cast and BeeMed. He owns stock options in Follow Health. He is on the board of the French Arthroscopic Society. Philippe Collin receives royalties from and is a consultant and paid speaker for Stryker and Enovis. He is the co‐founder of Med4Cast and Follow. He is on the board of SECEC and IBSES. The remaining authors declare no conflict of interest.

## ETHICS STATEMENT

The authors have nothing to report.

## Supporting information

Supplementary Table 1

Supplementary Table 2

Supplementary Table 3

Supplementary Table 4

Supplementary Table 5

Supplementary Table 6

Supplementary Table 7

## Data Availability

The data that support the findings of this study are available from the corresponding author upon reasonable request.
