# Supplementary material for: Moderate to high levels of spin in studies comparing open and arthroscopic Latarjet procedures for anterior shoulder instability: A systematic review from the LaTour Group
Source: Knee Surg Sports Traumatol Arthrosc. 2026 Jun 15;34(10):3732–45. doi: 10.1002/ksa.70476 (PMC13622913; doi:10.1002/ksa.70476)
Supplement: Supplementary file 1 [file KSA-34-3732-s001.docx]

Supplementary Table 2: Prevalence of spin stratified by level of evidence. Shows the spin ratio per study across Level II, III, and IV evidence.

| **Level of Evidence** | **Number of studies (total 25)** | **Prevalence of spin(count)** | | **Spin ratio per study** |
| --- | --- | --- | --- | --- |
| II | 3 | 3 |  | 1,00 |
| III | 18 | 21 |  | 1,17 |
| IV | 4 | 8 |  | 2,00 |
