# Supplementary material for: Moderate to high levels of spin in studies comparing open and arthroscopic Latarjet procedures for anterior shoulder instability: A systematic review from the LaTour Group
Source: Knee Surg Sports Traumatol Arthrosc. 2026 Jun 15;34(10):3732–45. doi: 10.1002/ksa.70476 (PMC13622913; doi:10.1002/ksa.70476)
Supplement: Supplementary file 2 [file KSA-34-3732-s002.docx]

**Supplementary Table 7.** Spin prevalence by publication year.

| **Publication date** | **Number of studies (total 25)** | **Prevalence of spin** |  | **Spin ratio per study** |
| --- | --- | --- | --- | --- |
| **2023-2025** | **9** | **11** |  | **1,22** |
| **2020-2022** | **5** | **4** |  | **0,80** |
| **2017-2019** | **8** | **7** |  | **0,88** |
| **up to 2016** | **3** | **7** |  | **2,33** |
