# Supplementary material for: Moderate to high levels of spin in studies comparing open and arthroscopic Latarjet procedures for anterior shoulder instability: A systematic review from the LaTour Group
Source: Knee Surg Sports Traumatol Arthrosc. 2026 Jun 15;34(10):3732–45. doi: 10.1002/ksa.70476 (PMC13622913; doi:10.1002/ksa.70476)
Supplement: Supplementary file 3 [file KSA-34-3732-s003.docx]

**Supplementary Table 6.** Spin prevalence by risk of bias.

| **Risk of Bias assessment*** | **Number of studies (total 25)** | **Prevalence of spin** |  | **Spin ratio per study** |
| --- | --- | --- | --- | --- |
| **80%-100%** | **8** | **9** |  | **1,13** |
| **60%-79%** | **13** | **15** |  | **1,15** |
| **40%-59%** | **2** | **4** |  | **2,00** |
| **20%-39%** | **1** | **2** |  | **2,00** |
| **0%-19%** | **1** | **2** |  | **2,00** |
| ***The percentage was calculated by assessing the frequency of 'Yes' in Table 2 and 3, compared to the maximum possible score (higher percentage equated to lower risk of bias)** | | | | |
