# Supplementary material for: Moderate to high levels of spin in studies comparing open and arthroscopic Latarjet procedures for anterior shoulder instability: A systematic review from the LaTour Group
Source: Knee Surg Sports Traumatol Arthrosc. 2026 Jun 15;34(10):3732–45. doi: 10.1002/ksa.70476 (PMC13622913; doi:10.1002/ksa.70476)
Supplement: Supplementary file 4 [file KSA-34-3732-s004.docx]

Supplementary Table 1: Prevalence and types of spin identified across 25 studies. Organized by spin category: misleading reporting, misleading interpretation, and inadequate extrapolation.

| **Category of spin** | **Strategy used** | **Number of studies** | **Prevalence**  **(%)** |
| --- | --- | --- | --- |
| **At least one spin in the conclusions** |  | 20 | (80%) |
| **Misleading reporting** | Not reporting adverse events or lack of focus on harm | 2 | (8%) |
|  | Selective reporting | 12 | (48%) |
|  | Misleading description of study design | 0 | 0% |
|  | Use of linguistic spin | 0 | 0% |
|  | No consideration of the limitations | 5 | (20%) |
|  | Selective citation of other studies | 0 | 0% |
| **Inadequate interpretation** | Claim an effect for non-statistically significant results | 1 | (4%) |
|  | Claim an equivalence for non-statistically significant results despite a wide confidence interval | 2 | (8%) |
|  | Ruling out safety for non-statistically significant results | 0 | 0% |
|  | Causal language or causal claim | 2 | (8%) |
|  | Claim of any significant difference despite lack of statistical test | 5 | (20%) |
|  | Focus on statistical significance instead of clinical relevance | 0 | 0% |
| **Inadequate extrapolation** | Inadequate extrapolation to larger population, intervention or outcome | 2 | (8%) |
|  | Inadequate implication for clinical practice | 1 | (4%) |
