# Supplementary material for: Moderate to high levels of spin in studies comparing open and arthroscopic Latarjet procedures for anterior shoulder instability: A systematic review from the LaTour Group
Source: Knee Surg Sports Traumatol Arthrosc. 2026 Jun 15;34(10):3732–45. doi: 10.1002/ksa.70476 (PMC13622913; doi:10.1002/ksa.70476)
Supplement: Supplementary file 5 [file KSA-34-3732-s005.docx]

Supplementary Table 4: Prevalence of spin based on funding disclosure.

| **Study funding** | **Number of studies (total 25)** | **Prevalence of spin(count)** | | **Spin ratio per study** |
| --- | --- | --- | --- | --- |
| Yes declared (with funding) | 0 | 0 |  |  |
| Yes declared (without funding) | 7 | 9 |  | 1,29 |
| Not reported | 18 | 23 |  | 1,28 |
