# Supplementary material for: Moderate to high levels of spin in studies comparing open and arthroscopic Latarjet procedures for anterior shoulder instability: A systematic review from the LaTour Group
Source: Knee Surg Sports Traumatol Arthrosc. 2026 Jun 15;34(10):3732–45. doi: 10.1002/ksa.70476 (PMC13622913; doi:10.1002/ksa.70476)
Supplement: Supplementary file 6 [file KSA-34-3732-s006.docx]

Supplementary Table 3: Prevalence of spin based on conflict-of-interest reporting.

| **Conflict of interest** | **Number of studies (total 25)** | **Prevalence of spin(count)** | | **Spin ratio per study** |
| --- | --- | --- | --- | --- |
| Yes declared (with conflict of interest) | 12 | 15 |  | 1,25 |
| Yes declared (without conflict of interest) | 9 | 11 |  | 1,22 |
| Not reported | 4 | 6 |  | 1,50 |
