# Supplementary material for: Moderate to high levels of spin in studies comparing open and arthroscopic Latarjet procedures for anterior shoulder instability: A systematic review from the LaTour Group
Source: Knee Surg Sports Traumatol Arthrosc. 2026 Jun 15;34(10):3732–45. doi: 10.1002/ksa.70476 (PMC13622913; doi:10.1002/ksa.70476)
Supplement: Supplementary file 7 [file KSA-34-3732-s007.docx]

Supplementary Table 5: Prevalence of level of spin identified in included studies.

| **Level of spin** | **Number of studies (total 25)** | **Prevalence of spin(count)** | | **Spin ratio per study** |
| --- | --- | --- | --- | --- |
| High | 11 | 18 |  | 1,64 |
| Moderate | 5 | 10 |  | 2,00 |
| Low | 4 | 4 |  | 1,00 |
